# Editorial: Congenital obstruction of the urinary tract

**DOI:** 10.3389/fped.2025.1626486

**Published:** 2025-05-30

**Authors:** Irene Paraboschi, Massimo Garriboli

**Affiliations:** ^1^ Department of Biomedical and Clinical Sciences, University of Milano, Milano, Italy; ^2^ Department of Pediatric Surgery, “V. Buzzi” Children’s Hospital, Milano, Italy; ^3^ Department of Pediatric Urology, Evelina London Children’s Hospital, Guy’s and St Thomas’ NHS Foundation Trust, London, United Kingdom

**Keywords:** pediatric urology, obstructive uropathies, urinary tract obstruction, prevention, early diagnosis

**Editorial on the Reaserch Topic**
Congenital obstruction of the urinary tract

The landscape of pediatric urology continues to evolve in response to the rising demand for tailored surgical strategies, refined diagnostic accuracy, and the integration of molecular and imaging-based technologies. This Research Topic brings together five high-quality studies—four original research articles and one case report—each offering critical insights into rare congenital urological anomalies, minimally invasive surgical techniques, and novel translational diagnostics.

Beanman et al. present a clinically exceptional case of prolonged survival in a child with Schinzel–Giedion Syndrome (SGS), characterized by a SETBP1 mutation and primary non-refluxing megaureter (Beanman et al.). Notably, the patient, still alive at 11 years of age, surpasses the typical early mortality associated with SGS. The authors demonstrate expression of SETBP1 in the fetal urinary tract using immunohistochemistry, thus supporting its role in lower urinary tract development. This study reinforces the value of early exome sequencing in syndromic presentations of congenital anomalies. It highlights the emerging relevance of neurodevelopmental-genitourinary overlap in REOLUT (rare early-onset lower urinary tract) disorders.

In a large retrospective cohort, Wong and Tam assess the utility of intraoperative cystoscopy to identify prostatic utricle cysts (PUC) in patients with proximal hypospadias (Wong and Tam). Their findings demonstrate a 74% prevalence of PUC, with cysts ≥20 mm significantly associated with medium-term symptomatic sequelae, including post-void dribbling and epididymoorchitis. These results point toward a potential paradigm shift, where concurrent cystoscopy during hypospadias repair could become standard in selected cases, particularly those with differences of sex development (DSD) or ambiguous genitalia. Fluorescence-enhanced visualization may further refine the detection of such anomalies in the future (1).

Mandaletti et al. report on a well-characterized multicenter cohort of fetuses diagnosed prenatally with megacystis, highlighting the limitations of current prognostic tools for postnatal renal function (Mandaletti et al.). Despite analyzing a wide range of sonographic parameters (including bladder diameter, keyhole sign, and gestational age at diagnosis), no single factor could reliably predict renal deterioration. Their findings echo conclusions from recent systematic reviews, which stress the inconsistency of existing imaging and biochemical predictors (2). This underscores the critical need for multiparametric fetal risk stratification systems, ideally incorporating advanced imaging modalities such as fetal diffusion-weighted MRI or urinary proteomics (3).

From a translational viewpoint, Wang et al. explore the role of protease-activated receptors (PAR1 and PAR2) in the stenotic segments of the ureteropelvic junction (UPJ) in children with congenital obstruction (Wang et al.). Their immunofluorescence and RT-PCR results suggest that PAR2, which is significantly downregulated in UPJO tissues, may regulate SIP syncytium function and smooth muscle contractility. These molecular insights pave the way for future research into pharmacologic modulation of neuromuscular signaling to restore peristalsis in functional obstructions, potentially complementing findings from earlier biomarker studies on obstructive nephropathy (3).

The final case by Osipov et al. documents a bilateral Cowper's syringocele in an adolescent—a rare condition—diagnosed through MRI, urethrography, and urethroscopy (Osipov et al.). Surgical marsupialization under endoscopic and laser guidance achieved complete resolution of post-micturition dribbling. The intraoperative diagnostic approach and favorable functional outcome highlights the increasing role of real-time imaging and precision-guided surgery in pediatric urology. In the future, fluorescence-guided surgery (FGS) may play an adjunctive role in such reconstructions, as described in hypospadias and exstrophy repair (4, 5).

These contributions illustrate the multidimensional progress underway in pediatric urology—from fetal imaging to intraoperative fluorescence-enhanced guidance and from genetic profiling to neuromuscular signaling pathways. The Research Topic highlights the importance of integrating fundamental research and technical innovation into clinical practice. Continued collaboration between geneticists, radiologists, surgeons, and bioengineers will be essential to sustain this momentum and ensure a more effective, personalized, and preventative care for future generations of pediatric patients.
